# The impact of non-alcoholic fatty liver disease on sleep apnea in healthy adults: A nationwide study of Korea

**DOI:** 10.1371/journal.pone.0271021

**Published:** 2022-07-20

**Authors:** Namkyun Kim, Jae-Hyung Roh, Hanbyul Lee, Doyeon Kim, Sung Jae Heo

**Affiliations:** 1 Department of Internal Medicine, School of Medicine, Kyungpook National University, Daegu, Republic of Korea; 2 Department of Cardiology in Internal Medicine, School of Medicine, Chungnam National University Sejong Hospital, Chungnam National University, Sejong, Korea; 3 Division of Healthcare Business Development, Chungnam National University Sejong Hospital, Sejong, Korea; 4 Department of statistics, Kyungpook National University, Daegu, Korea; 5 Department of Otorhinolaryngology-Head and Neck Surgery, School of Medicine, Kyungpook National University, Daegu, Republic of Korea; Sapienza University of Rome, ITALY

## Abstract

**Background & aims:**

Nonalcoholic fatty liver disease (NAFLD) is one of the most common health problems worldwide. Sleep apnea (SA) causes cardiovascular and metabolic problems, as well as a significant socioeconomic burden. Although several studies have found that SA causes NAFLD, there is no evidence that NAFLD causes SA. The goal of this study was to look at the relationship between NAFLD and SA in realworld data.

**Methods:**

We evaluated 334,334 healthy individuals without comorbidities who underwent National Health checkups in the Republic of Korea from 2009 to 2014. NAFLD was defined by a surrogate marker, the fatty liver index (FLI). The association between FLI and SA was analyzed using multivariate Cox proportional hazards regression models.

**Results:**

During a median followup of 5.3 years, 1,351 patients (0.4%) were newly diagnosed with SA. Subjects were categorized into quartile groups according to FLI (range: Q1, 0–4.9; Q2, 5.0–12.5; Q3, 12.6–31.0; Q4, >31.0). Subjects with higher FLIs had a significantly higher cumulative incidence of SA than those with lower FLIs (Q1, 119 [0.1%]; Q2, 210 [0.3%]; Q3, 339 [0.4%]; Q4, 683 [0.8%]; *P* < 0.001). Adjusted hazard ratios (HRs) revealed that a higher FLI was independently associated with an increased risk of SA (HR between Q4 and Q1, 4.03; 95% confidence interval, 3.22–5.05; *P* < 0.001). This association remained statistically significant after further adjustment for Body mass index (BMI) (HR between Q4 and Q1, 2.19; 95% confidence interval, 1.69–2.83; *P* < 0.001). FLI was significantly associated with an increased risk of new-onset SA regardless of baseline characteristics.

**Conclusion:**

This study demonstrated that NAFLD, assessed by FLI, was independently associated with increased risk for SA in the healthy Korean population.

## Introduction

Nonalcoholic fatty liver disease (NAFLD) is one of the most common health problems worldwide. Because NAFLD is typically a silent disease, exact incidence rates are unknown; however, it is commonly estimated to be in the range of 27%–34% in the general population [1]. Obesity, diabetes mellitus, and metabolic syndrome are all associated with the prevalence of NAFLD. It is estimated that 75%–92% of morbidly obese patients and 60%–70% of type 2 diabetes patients are affected [1, 2]. Sleep apnea (SA) is a variety of respiratory diseases including obstructive sleep apnea (OSA) and central sleep apnea that cause nocturnal hypoxemia. OSA is a heterogeneous disorder characterized by repetitive narrowing or closure of the upper airway during sleep. It affects 2%–4% of the general population and 35%–45% of obese people [3, 4]. OSA can lead to systemic hypertension, cognitive decline, and even permanent brain damage [5, 6]. However, since the hypertension and decreased cognitive function can be improved with OSA treatment, caution is required for OSA [7].

Although several studies are showing a positive association between SA and NAFLD using fatty liver index (FLI) or histologic examination, they are small sample sizes and showed a simple association in several cross-sectional studies [8, 9]. Previous studies focused only on the occurrence of NAFLD due to hypoxic damage in SA. Liver inflammation is caused by the interaction of various inflammatory factors including hypoxic damage [10]. Thus, we examined a large, comprehensive cohort of healthy Korean adults who lacked traditional risk factors and comorbidities to determine the direct relationship between NAFLD and SA.

## Methods

### Data source

We analyzed the National Health Insurance Service–National Sample Cohort 2.0 (NHIS-NSC 2.0) data set which is the population-based sample cohort data of Korea. The NHIS-NSC 2.0 was made by extracting random samples of about 2% of the total Korean population in 2006 with retrospective and prospective followup data from 2002 to 2015. Koreans are required by law to obtain government-provided National Health insurance, and NHIS covers 97% of Koreans. The Korean government stratified insured population (n = 48,222,537) into 2,142 classes according to their by gender, age, region, eligibility status, and income level. The Korean government then randomly selected 2.1% from each stratum (n = 1,021,208). As a result, the NHIS-NSC 2.0 cohort can represent the entire Korean population [11].

The cohort contains four databases: the first is a sociodemographic information dataset of the beneficiaries; the second is a medical claims dataset with diagnoses based on the 10th revision of the International Classification of Disease (ICD-10) codes, admission, and treatment; the third is a National Health Screening dataset of the cohort members; and the fourth is a medical institutions dataset.

Korean adults over the age of 20 are recommended the National Health checkup biennially, which is composed of blood tests, chest X-rays, physical examinations, and questionnaires on medical history and health-related behaviors including smoking status and alcohol consumption. According to NHIS data from 2013, 72.1% of eligible beneficiaries received a National Health checkup [7]. The cohort also included mortality data such as the date and cause of death from the National statistical office’s death registration database, which is a government statistical agency.

All researchers whose protocols have been approved by the NIHS review board can use the NHIS-NSC 2.0. The Chungnam National University Hospital’s Institutional Review Board in Daejeon, Korea, approved this study and waived the requirement for informed consent.

### Study population

This study included a population over 20 years of age who received National Health checkups at least once from 2009 to 2014. The index checkup was defined as the first National checkup, and the index year was the year of the index checkup. Subjects who met the predefined exclusion criterion were excluded.: (a) those suffering from congestive heart failure, hypertension, diabetes, cerebrovascular disease, ischemic heart disease, peripheral artery disease, or liver disease; (b) those with rheumatic or nonrheumatic valvular heart disease; (c) those who with increased baseline SBP ≥140 mmHg or DBP ≥90 mmHg, and fasting blood glucose ≥126 mg/dL at the index checkups; and (d) those found to have missing data in the index checkup. Each diagnosis was based on 1-year clams data before the index year or index checkups (the questionnaires for medical history, and measurement of blood pressure and fasting glucose levels). We defined each diagnosis in the claims data as the first occurrence on at least two different days of hospital visits (outpatient) or on the first admission. The ICD-10 codes are used to define each diagnosis.

### Definition of data

Smoking status was categorized into ex- or current smokers, and nonsmokers. Alcohol consumption was assessed using standardized self-reporting questionnaires that included questions about how many days of the week alcohol is consumed and how much alcohol is consumed on each drinking day. The amount of alcohol consumption was calculated by multiplying them. Body mass index (BMI) was calculated by dividing weight (kg) by height squared (m^2^). The physical activity questionnaires included questions about how many days a week 30 minutes of light exercise, 30 minutes of moderate exercise, and 20 minutes of vigorous exercise are performed, respectively. The light exercise was assumed to be 2 metabolic equivalent of tasks (METs), moderate 3METs, and vigorous 6METs, which were multiplied by 20, 30, and 30 minutes and the respective number of days a week, and summed.

The primary outcome of interest was finding the incidence of SA. The first occurrence of SA during at least two different days of outpatient visits, admission for SA, or death with a diagnosis of SA was defined as the SA incidence. Data were censored at the time of SA occurrence, NHIS disqualification (death or immigration), or study completion (December 31th, 2015).

### Diagnosis of steatosis

A well-validated, surrogate marker, FLI was used to identify patients with steatosis [12]. FLI was composed of four variables (triglycerides [TG], Body mass index [BMI], gamma-glutamyltransferase [GGT], and waist circumference [WC] and calculated as follows:

$$FLI=(e^{0.953\times loge(TG)+0.139\times BMI+0.718\times loge(GGT)+0.053\times WC-15.745})/(1+e^{0.953\times }{}^{loge(TG)+0.139\times BMI+0.718\times loge(GGT)+0.053\times WC-15.745})\times 100.$$

The original study suggested an FLI ≥ 60 as the cutoff for a diagnosis of hepatic steatosis with a positive likelihood ratio of 4.3 in the general population [12]. Although the FLI is simple to obtain and calculate for screening fatty liver disease, there is insufficient evidence to support the diagnosis of fatty liver disease in Asians with the FLI due to lower BMI and WC than other ethnic populations [13]. Resultantly, we categorized our study population into quartiles according to their FLI value and used the quartile group in the statistical analysis.

### Statistical analysis

Data are presented as mean ± standard deviation for continuous variables and as number (percentage) for categorical variables. The differences between FLI quartiles were compared using oneway analysis of variance and chi-square tests. The incidence of SA was calculated by dividing the total number of newly diagnosed SA cases between 2009 and 2014 by the total population during the same period and expressing the result as cases per 100,000 person-years. Cumulative event rates were calculated and compared between FLI quartile-based groups using Kaplan–Meier estimates and the log-rank test. For SA incidence, Cox proportional hazards regression was used to estimating adjusted hazard ratios (HRs), and 95 percent confidence intervals (CIs) were used. Data were first adjusted for age and gender (model 1). Then, clinical characteristics of which association with new-onset SA demonstrates borderline statistical significance (P < 0.10), smoking, alcohol consumption, activity, systolic blood pressure, diastolic blood pressure, fasting glucose, and low-density lipoprotein cholesterol were incorporated into model 1 to obtain model 2. In model 3, multivariate analysis was performed, and BMI, which is an important risk factor for both NAFLD and SA, was added to model 2. The variance inflation factor was used to test the multicollinearity of the variables included in the analysis, and all of them were less than 10, indicating that they had no effect on the results.

For subgroup analyses, FLI was integrated into the statistical models as a continuous variable after log transformation. A two-sided *P* value of <0.05, was considered to indicate statistical significance. R software, version 3.3.3, was used for statistical analyses (R Foundation for Statistical Computing, Vienna, Austria; www.r-project.org).

## Results

### Baseline characteristics (of the participants)

**Fig 1:**
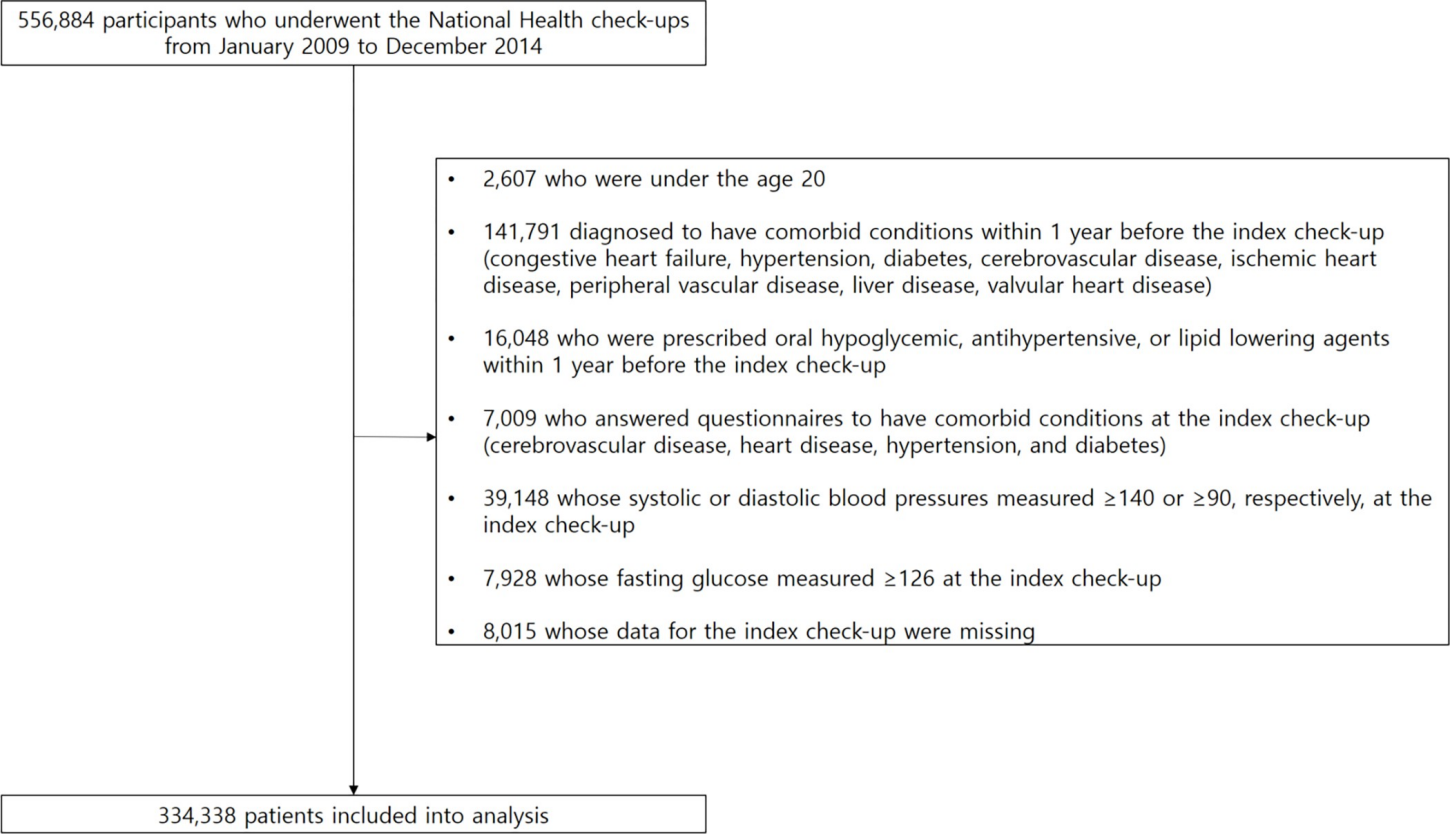
Overview of the study population.

Of the total 556,884 NHIS-NSC 2.0 sample cohort population, the exclusion of subjects who met the prespecified exclusion criteria left 334,334 subjects into the analysis. Fig 1 shows a list of subjects who meet each exclusion criterion. According to FLI quartile values, the study population was divided into four groups (first quartile [Q1], 0–4.9; second quartile [Q2], 5.0–12.5; third quartile [Q3], 12.6–31.0; and fourth quartile [Q4], >31.0). Table 1 summarizes the baseline clinical characteristics of the study population based on the FLI quartile. The subjects with higher FLI were older and more likely to be male and obese than those with a lower FLI. From Q1 to Q4, the proportion of current smokers and the amount of alcohol consumed were expected to rise. Subjects with higher FLIs had higher blood pressure, fasting blood glucose, and GGT levels than those with lower FLIs. With increasing FLI values, the lipid profile becomes increasingly unfavorable.

**Table 1:** Baseline characteristics.

	FLI quartile				*P* value
	Q1	Q2	Q3	Q4	
	(n = 83,584)	(n = 83,575)	(n = 83590)	(n = 83585)	
Age (year)	37.0 ± 11.6	42.3 ± 12.5	44.4 ± 12.6	43.4 ± 11.5	<0.001
Male (%)	12,831 (15.4)	32,489 (38.9)	49,372 (59.1)	66,646 (79.7)	<0.001
BMI (kg/m^2^)	20.0 ± 1.8	22.1 ± 1.8	23.8 ± 2.1	26.3 ± 2.8	<0.001
Waist circumference (cm)	67.9 ± 4.9	74.9 ± 4.8	80.5 ± 5.1	87.6 ± 6.5	<0.001
Systolic BP (mmHg)	110.9 ± 11.1	115.4 ± 11.1	118.6 ± 10.6	121.8 ± 10.0	<0.001
Diastolic BP (mmHg)	69.4 ± 7.9	71.9 ± 8.0	74.0 ± 7.6	76.3 ± 7.1	<0.001
Smoking					<0.001
Nonsmoker	70,774 (84.7)	58,765 (70.3)	47,126 (56.4)	31584 (37.8)	
Ex-smoker	3,931 (4.7)	7,261 (8.7)	11,552 (13.8)	15,164 (18.1)	
Current smoker	8,879 (10.6)	17,549 (21.0)	24,912 (29.8)	36,837 (44.4)	
Alcohol consumption (g/wk)	31.1 ± 76.6	48.3 ± 101.0	71.6 ± 127.9	120.0 ± 173.0	<0.001
Activity (MET-min/wk)	346.3 ± 351.6	379.2 ± 383.3	388.0 ± 389.0	373.8 ± 374.9	<0.001
Laboratory findings					
Fasting glucose (mg/dL)	87.9 ± 9.5	90.3 ± 10.2	92.2 ± 10.8	94.8 ± 11.4	<0.001
Total cholesterol (mg/dL)	177.0 ± 34.0	187.3 ± 35.8	196.5 ± 39.1	207.0 ± 40.3	<0.001
LDL cholesterol (mg/dL)	108.9 ± 247.6	113.7 ± 141.8	121.2 ± 129.3	122.5 ± 124.7	<0.001
HDL cholesterol (mg/dL)	64.1 ± 24.4	59.3 ± 20.0	55.6 ± 26.1	51.7 ± 32.0	<0.001
Triglyceride (mg/dL)	62.7 ± 22.6	88.2 ± 34.0	119.9 ± 53.5	196.7 ± 130.7	<0.001
GGT (U/L)	14.2 ± 5.6	19.1 ± 9.9	27.9 ± 20.3	59.7 ± 61.8	<0.001
Creatinine (mg/dL)	0.9 ± 1.0	1.0 ± 1.1	1.0 ± 1.2	1.1 ± 1.2	<0.001

Data expressed as mean ± standard deviation or number (%)

FLI = fatty liver index; BMI = body mass index; BP = blood pressure; MET = metabolic equivalent of task; LDL = low-density lipoprotein; HDL = high-density lipoprotein; GGT = γ-glutamyltransferase

### Association between FLI and the incidence of SA

**Fig 2:**
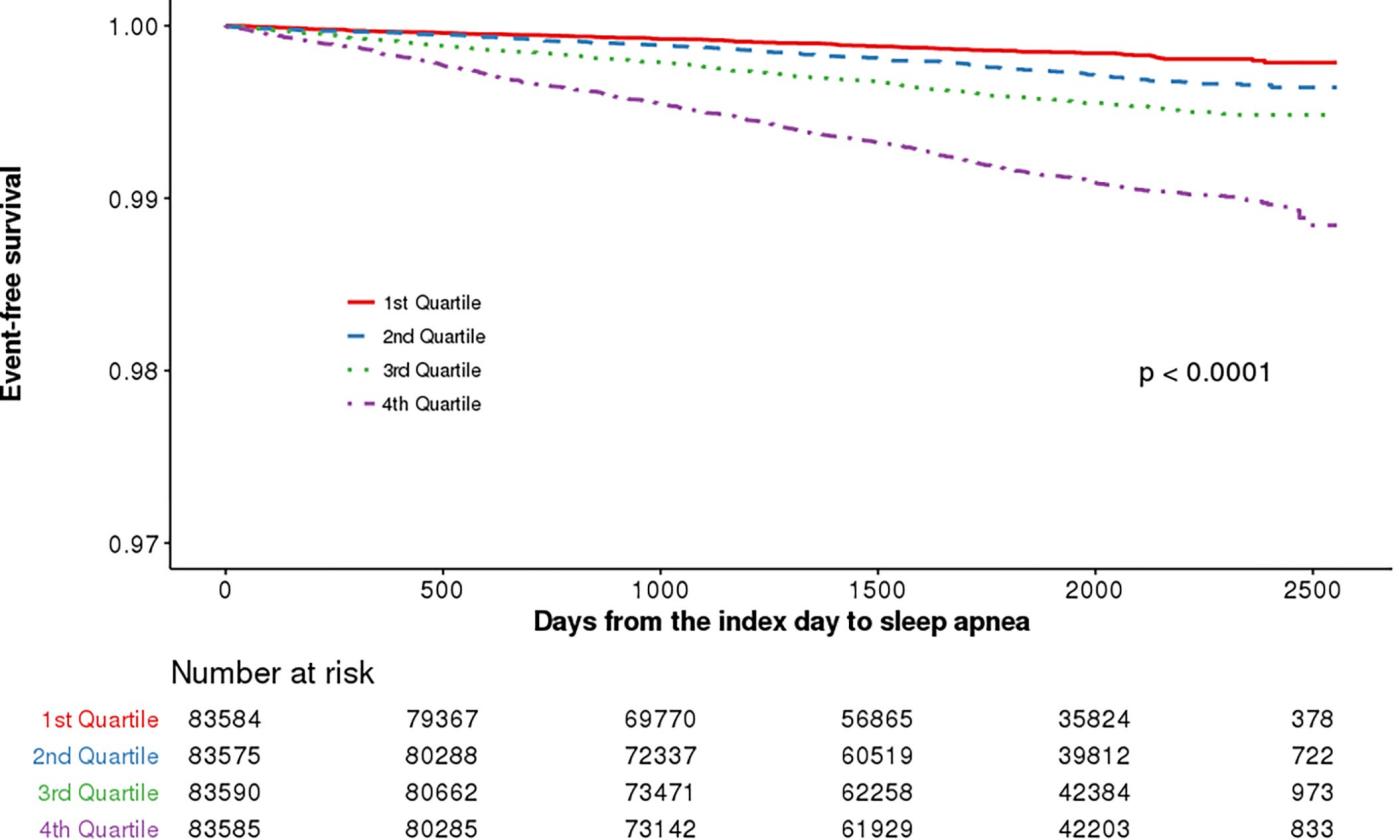
Cumulative incidence of new-onset sleep apnea (SA), according to their quartiles.

The followup duration was a median of 5.3 years (interquartile range, 4.1–6.3), during which 1,351 subjects (0.4%) developed new-onset SA. Fig 2 depicts the cumulative incidence of new-onset SA according to FLI quartile groups. When comparing subjects with higher FLIs to those with lower FLIs, the incidence of SA was significantly higher in those with higher FLIs (Q1, 119 [0.1%]; Q2, 210 [0.3%]; Q3, 339 [0.4%]; Q4, 683[0.8%]). In multivariate models which were adjusted for age and gender (model 1), the HR (95% CI) was 3.67 (2.95–4.57) for the highest group of FLI compared with that of the lowest. When the model was further adjusted for clinical characteristics with *P* < 0.10 in the univariate analyses (model 2), the HR (95% CI) was 4.03 (3.22–5.05) for the highest group of FLI compared with that of the lowest. The relationship between FLI quartile and SA incidence remained statistically significant after adjusting for BMI in model 2 (Table 2).

**Table 2:** Association between fatty liver index and new-onset sleep apnea.

FLI quartile	Total (N)	Event (n, %)	Univariate			Model 1^a^		
			HR	95% CI	*p* value	HR	95% CI	*p* value
Q1	83,584	119 (0.1)	Reference			Reference		
Q2	83,575	210 (0.3)	1.70	1.35–2.12	<0.001	1.47	1.17–1.85	0.001
Q3	83,590	339 (0.4)	2.68	2.18–3.30	<0.001	2.07	1.65–2.58	<0.001
Q4	83,585	683 (0.8)	5.42	4.46–6.59	<0.001	3.67	2.95–4.57	<0.001
FLI quartile			Model 2^b^			Model 3^c^		
			HR	95% CI	*p* value	HR	95% CI	*p* value
Q1			Reference			Reference		
Q2			1.52	1.20–1.91	<0.001	1.24	0.98–1.56	0.079
Q3			2.18	1.74–2.73	<0.001	1.50	1.18–1.90	0.001
Q4			4.03	3.22–5.05	<0.001	2.19	1.69–2.83	<0.001

FLI = fatty liver index; HR = hazard ratio; CI = confidential interval; BMI = body mass index

^a^Cox proportional hazard models including age, and gender as covariates

^b^Cox proportional hazard models including age, gender, smoking, alcohol consumption, activity, systolic blood pressure, diastolic blood pressure, fasting blood glucose, and low-density lipoprotein as covariates

^c^Cox proportional hazard models including BMI, in addition to the covariates incorporated in Model 2.

In addition to the FLI groups, the analysis was carried out after categorizing the study subjects according to the various FLI cutoff criteria proposed in previous studies. Regardless of the cutoff-points used, the highest FLI goup had the highest risk for new onset SA.

### Subgroup analysis (of clinical variable affecting SA incidence)

**Fig 3:**
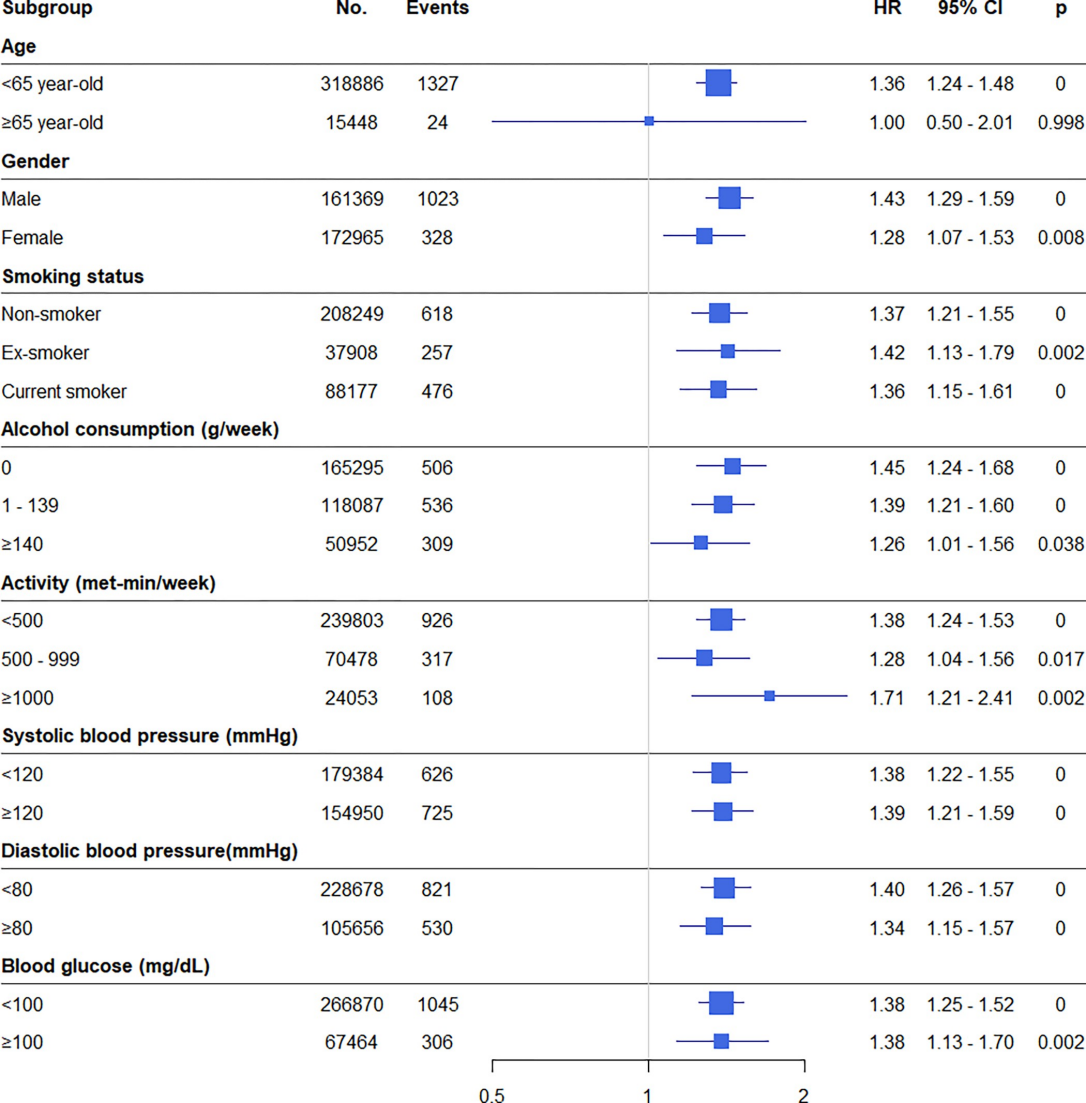
Forest plots of hazard ratios for new-onset SA stratified by various clinical characteristics. HR, hazard ratio; CI, confidence interval.

Adjusted HRs according to subgroups summarized in Fig 3. FLI was a significant determinant of new-onset SA in all subgroups (Fig 3).

## Discussion

In this study, we looked at the relationship between NAFLD as measured by FLI, a well-validated surrogate biomarker, and the prevalence of SA in a large, realworld data set from the Korean population. Recent studies demonstrate an association between NAFLD and SA [8, 14, 15]. Krolow et al. analyzed 51 patients and reported significant associations between histologically proven liver fibrosis and moderate to severe OSA [8]. With 153 participants who had NAFLD confirmed by liver ultrasound, Zhang et al. demonstrated that OSA plays a role in liver injury among severely obese individuals [14]. Wei et al. reported that patients with a sleep disorder had a higher risk of developing NAFLD, including those sleep disorder patients who did not have SA [16]. Previous studies, however, had limitations in that the sample size was too small [5, 10], the subjects were limited to severely obese patients [10], or it was a meta-study [11]. Furthermore, there is limited evidence that fatty liver is directly related to the occurrence of SA. In this study, realworld data revealed that the incidence of SA was high in patients with fatty liver.

Several mechanisms potentially link NAFLD and SA beyond the shared risk factors. Firstly, the wider neck circumference in NAFLD patients than in the general population may cause SA. NAFLD patients have a wider neck circumference (NC) than healthy subjects, according to previous research [13]. Kim et al. demonstrated that the NC can predict both the presence and severity of OSA [17]. Secondly, patients with NAFLD have a high incidence of asthma [18], and asthma and allergic rhinitis are closely related [19]. Furthermore, given the high prevalence of SA in patients with allergic rhinitis [17], it may explain why patients with NAFLD have more SA. Finally, NAFLD may be associated with increased systemic inflammation [18], which may lead to SA. Excess triglyceride accumulation in hepatocytes has been linked to impaired fatty acid oxidation, increased oxidative stress, and local inflammation, all of which can potentially lead to the progression from simple steatosis to steatohepatitis [20]. Previous studies have found that the degree of hepatic steatosis and inflammation correlated with systemic levels of inflammatory biomarkers including C-reactive protein (CRP) [21]. According to Gaines et al., patients with elevated CRP are more likely to develop SA [22]. The oxidative stress associated with NAFLD is also closely related to SA [23]. As the prevalence of NAFLD, which is potentially associated with SA, is increasing, attention should be paid to SA as well as NAFLD [24].

Given the observed link between NAFLD and SA, as well as the mechanisms underlying this link, it is reasonable to assume that interventions for NAFLD management may have the therapeutic potential to reverse the natural course of SA. Obesity is widely recognized as a major risk factor for SA, and if achieved, significant and sustained weight loss is likely to be a useful therapeutic option in the management of SA [25]. Because NAFLD is also strongly linked with obesity and has been shown to improve with weight loss [26], the observed beneficial effect of weight loss on the natural progression of NAFLD may be partly attributed to the improvement of SA. Many therapeutic interventions other than weight loss are under intense investigation for the management of NAFLD. They include bariatric surgery, treatment with thiazolidinediones, glucagon-like peptide-1 receptor agonists and sodium–glucose cotransporter-2 inhibitors, all of which showed a promising effect against NAFLD [26]. The impact of these upstream interventions aimed at NAFLD on the course of SA disease must be studied further in the future. Despite significant correlations between hepatic steatosis and SA severity markers, continuous positive airway pressure alone did not improve hepatic steatosis or fibrosis. However, the additional role of weight reduction through lifestyle modification deserves further investigation [27].

Several limitations of this study need to be acknowledged. To begin, we estimated new-onset SA using the NHIS-NSC 2.0 database, which is based on ICD-10 disease codes; however, the accuracy of SA diagnosis was not validated in this study. More research is needed to validate SA patient data with health insurance data using hospital-based medical records. Second, steatosis was determined using a surrogate biochemical marker rather than histology or magnetic resonance spectroscopy. An invasive procedure or a complex, expensive imaging modality, on the other hand, is not feasible in large cohorts. FLI, on the other hand, is a widely accepted surrogate for hepatic steatosis that has been validated in the general population [12]. Interestingly, an increased risk for new-onset SA was observed in the subjects with FLI values of around 30, which was much lower than the cutoff point suggested by Bedogni et al [12]. These findings back up Yang et al.’s study, in which they proposed lower cutoff points for Asians with different anthropometric characteristics than Westerns [28]. Finally, the study population’s exclusivity to Koreans may limit the current study’s generalizability to other ethnic populations.

In conclusion, our study demonstrated that NAFLD, as measured by FLI, was independently associated with increased risk for SA in the healthy Korean population. Because management of SA remains a difficult clinical challenge, the identification of potentially modifiable risk factors may have important for clinicians. More research on the effect of NAFLD management on the incidence and severity of SA is required.
